# Progress of Disintegration of Polylactide (PLA)/Poly(Butylene Succinate) (PBS) Blends Containing Talc and Chalk Inorganic Fillers under Industrial Composting Conditions

**DOI:** 10.3390/polym13010010

**Published:** 2020-12-22

**Authors:** Sengül Tolga, Stephan Kabasci, Mona Duhme

**Affiliations:** Department of Circular and Biobased Plastics, Fraunhofer UMSICHT, Fraunhofer Institute for Environmental, Safety, and Energy Technology, Osterfelder Straße 3, 46047 Oberhausen, Germany; senguel.tolga@umsicht.fraunhofer.de (S.T.); stephan.kabasci@umsicht.fraunhofer.de (S.K.)

**Keywords:** poly(lactic acid), PLA, poly(butylene succinate), PBS, blend, inorganic filler, biodegradation behavior, disintegration, industrial composting conditions

## Abstract

Biodegradable plastics are experiencing increasing demand, in particular because of said property. This also applies to the two biopolyesters poly(lactic acid) (PLA) and poly(butylene succinate) (PBS) covered in this study. Both are proven to be biodegradable under industrial composting conditions. This study presents the influence of mineral fillers on the disintegration process of PLA/PBS blend systems under such conditions. Chalk and talc were used as fillers in PLA/PBS (7:3) blend systems. In addition, unfilled PLA/PBS (7:3/3:7) blend systems were considered. Microscopic images, differential scanning calorimetry and tensile test measurements were used in addition to measuring mass loss of the specimen to characterize the progress of disintegration. The mineral fillers used influence the disintegration behavior of PLA/PBS blends under industrial composting conditions. In general, talc leads to lower and chalk to higher disintegration rates. This effect is in line with the measured decrease in mechanical properties and melting enthalpies. The degrees of disintegration almost linearly correlate with specimen thickness, while different surface textures showed no clear effects. Thus, we conclude that disintegration in a PLA/PBS system proceeds as a bulk erosion process. Using fillers to control the degradation process is generally regarded as possible.

## 1. Introduction

Due to increasing criticism of today’s plastics usage, often referring to environmental pollution or a decline of petrochemical recourses, bioplastics, no matter if they are biodegradable or bio-based, have been receiving growing attention in recent decades. Particularly, biodegradable polymers such as poly(lactic acid) (PLA) or poly(butylene succinate) (PBS) are of high interest for the scientific community, bioplastic manufacturers and end-users. Simultaneously, the discussion of biodegradation of polymers, intermediate products and the overall impact is enhanced.

In general, there are different environmental conditions (compost, soil, water, in the human body, different temperatures and under aerobic or anaerobic influences) under which polymers degrade. According to the International Union of Pure and Applied Chemistry (IUPAC) definition, biodegradation means degradation caused by an enzymatic process resulting from the action of living cells [1].

Whenever biodegradation is investigated, denoting the conditions under which the experiments are performed is inevitable. The present paper describes the first step of degradation and the disintegration behavior of polymer blends under industrial composting conditions. This means that the experiments were carried out in close approach to a disintegration test (DIN EN ISO 16929) at 60 °C under aerobic conditions in a mature compost medium. The neat polymers under consideration (PLA and PBS) have been proven to be ultimately biodegradable under these conditions, which was evaluated by means of analyzing carbon dioxide production in respirometric experimental set-ups [2,3,4]. Thus, both polymers are considered as compostable. The goal of this study was to gain a better understanding of the disintegration behavior of polymer blends and of filled blend systems.

Both aliphatic polyesters, PLA and PBS, are available on a large scale. However, the neat polymers are of low interest due to insufficient properties for most applications. By inserting PBS into the PLA matrix, its high rigidity can be reduced. In reverse, PLA added as minor component enhances PBS toughness and strength [5,6,7,8]. The improvements gained using these binary blend systems, however, are not sufficient for most market applications. That is why many works on the investigation and improvement of PLA/PBS blend systems can be found. A brief overview of different approaches in the researchers’ society was given by Su et al. [8]. In this study, next to the use of PLA and PBS, the addition of the biodegradable polyester poly(butylene adipate-co-terephthalate) (PBAT) was also investigated.

Inorganic fillers are commonly added to thermoplastic polymers. As well as influencing their properties (e.g., mechanical properties such as higher strength or stiffness, and decreasing heat deflection or shrinkage for shortening cycle time periods during injection molding process), they often attract interest to achieve lower costs [9]. Strong interfacial bonds between the filler surface and matrix as well as a fine filler dispersion within the matrix material are important to achieve good reinforcement. Each recipe component affects the processing parameters and the compound properties, including the biodegradation behavior of the material.

Various works dealing with PBS or PLA in combination with fillers and their influence on degradation behavior have been published. Selected literature references are given in the following. The degradation of plastics is a consequence of polymer chain cleavage. This can be initiated in different ways—for example, by hydrolysis, thermal or mechanical activation as well as oxidation processes and also by enzymatic processes. Of these, hydrolysis, purely chemical or enzyme mediated, is the most important type of reaction for polyesters such as the PLA, PBS and PBAT used herein [10,11,12]. Biodegradation is defined as the decomposition of substances by living matter, mostly by microorganisms. Lucas et al. classified the process into three main steps:

1. biodeterioration (material fragmentation in tiny pieces due to decomposers such as microorganisms and/or abiotic factors)
2. depolymerisation/biofragmentation (molecular mass reduction due to chain cleavages)
3. mineralization and biomass production, sometimes named as “assimilation” (uptake of small molecules into the living cells and therein conversion to biomass, storage vesicles and metabolites, finally leading to total mineralization, which is the formation of simple molecules such as H_2_O and CO_2_) [12].

Many research works to determine the degradation behavior of PLA have been published. That is why it is well known that its degradation is often induced by a hydrolysis reaction. Water penetrates the material and generates chain cleavages in amorphous regions at the ester bonds [2,3,10,12,13,14,15,16,17]. Once initiated, the process is self-accelerating as a consequence of a high amount of carboxylic end groups being formed, which decrease the pH in the inner core of the PLA material [3,10]. The rate of hydrolytic degradation of PLA depends upon various factors: from the PLA structure itself (such as D-isomer content, grade of crystallinity, molecular mass, etc.) to the prevailing environmental conditions (such as temperature, pH or humidity and presence of fungi) [5,12]. As a consequence of hydrolysis, molecular mass decreases as do the mechanical properties. Later, microorganisms begin to digest oligomeric PLA chains. They can also affect degradation by excreting hydrolytic enzymes or by mechanical degradation—e.g., caused by growing fungi hypheae [12]. For other biodegradable polyester systems such as PBS, the same mechanisms are valid. Using polymer blends often leads to increased degradation rates due to the formation of dispersed particles of the minor component in the immiscible system [6,7,18]. At the interfaces, the mechanism of hydrolytic degradation starts [5,7].

Recently, studies about the influence of the polymer surface area and its structure and the biodegradation rate were published. Yang et al. evaluated various specimen shapes. They found that powder-shaped neat PBS and PLA degraded much faster than film-shaped samples. Independent of their thickness, the film specimens preserved their shapes and showed nearly the same slow biodegradation rates in compost with 65% moisture, at 58 °C for 50 to 60 days, whereas the powdered samples had a much faster degradation rate [4,19]. Chinaglia et al. showed that polybutylene sebacate (PBSe) ground to small sizes (50 µm) revealed higher degradation rates (at soil for 138 days and 28 °C) than larger powder samples (500 µm) [2,13].

Many investigations into PLA or PBS in combination with filler materials were conducted in the past [20]. The results provided helpful understanding between filler content and its influence on crystallization behavior [21]. Some research groups found an increase in the degradation rate of PLA by the use of clay minerals. Interfaces between filler and matrix are regarded as weak points where the degradation process begins [14,22].

Information about the degradation behavior of the neat biopolymers PLA and PBS as well as blends thereof are given in the literature. Many of the works deal with the degradation behavior after blend crosslinking. Furthermore, the general effects of organic filler materials on the blend properties have been presented [4,15,16,23,24,25]. The influence of filler type, size and amount on the degradation of PLA and PBS blends, however, has not been published yet. Loading blend systems with inorganic filler materials can lead to a faster degradation due to voids and increased hydrolysis at filler–matrix interfaces. On the other hand, fillers might slow down the degradation because of a higher hydrophobic character of the material or a higher crystallinity of the polymer. Therefore, this study investigates the disintegration behavior of PLA/PBS blends in combination with different quantities of talc and chalk as mineral fillers. The researchers’ choice fell on these two types of fillers, since they are most commonly used in thermoplastics. Of course, both types, chalk and talc, have different properties. For the current study, most important are the variations in shape: spherical chalk vs. flaky talc particles, and size: coarse and fine talc particles. Two assumptions prevailed the selection of the fillers. First, the smaller particles have a higher nucleation effect and the associated higher crystallinity might reduce the disintegration rate. Second, the platelet-shaped talc was assumed to hinder water penetration into the specimens, which reduces hydrolytically induced degradation.

Talc in two different particle sizes was used to elucidate the influence of filler size on the degradation rate. Mostly, PLA was used as the major component and PBS as the minor component. Further additives such as compatibilizers or coupling agents were not added. As well as the filler loaded blend systems, unloaded blend systems were evaluated as well. As well as PLA usage as major component, a PLA/PBS blend system with reversed major and minor components as well as a combination of PLA/PBAT, was tested. This served to evaluate the influence of the different polyester types and their quantities on the disintegration behavior. All polymers used, PLA, PBS, and PBAT, had proven to be compostable, which was determined by chemical analysis via measurement of CO_2_ production [3,4,26,27,28,29,30,31]. Such biodegradation measurements are not part of this work. Instead, the first stage of the degradation process, the disintegration behavior by fragmentation, was assessed for different blend types and different fillers as a comparative study.

It is well known that PLA requires a sufficiently high initial temperature to induce degradation. To ensure the degradability of PLA, the test specimens were subjected to industrial composting conditions. The samples were inspected after defined storage periods up to a maximum of 12 weeks. The mechanical properties of the samples were checked via tensile tests, they were characterized via differential scanning calorimetry, their appearance was inspected with regard to arising optical differences and the disintegration was measured based on the mass loss of the samples as single measurements. To determine the influence of sample thickness and surface roughness on the degradation, specimens of different thicknesses and with different surface structures were used as well.

## 2. Materials and Methods

### 2.1. Materials

Poly(lactic acid) (PLA) was purchased from Nature Works LLC, Minnetonka, MN, USA. The used grade—named Ingeo^TM^ 3251D with a D-isomer content of approximately 1.4%—has a density of 1.24 g/cm^3^. With a melt flow ratio (MFR) (190/2.16) of 35 g/10 min, it is designed for injection molding applications.

Poly(butylene succinate) (PBS) was used as grade BioPBS^TM^ FZ71PM from PTT MCC Biochem Co., Ltd., Bangkok, Thailand. It has a density of 1.26 g/cm^3^ and an MFR (190/2.16) of 22 g/10 min.

Poly(butylene adipate-co-terephthalate) (PBAT) ecoflex^®^ F Blend C1200 from BASF SE, Ludwigshafen, Germany is a statistical aliphatic-aromatic copolyester with a density of about 1.25 g/cm^3^ and an MFR (190/2.16) of 2.7 to 4.9 g/10 min. It is particularly suitable for blown film applications.

As inorganic filler materials, two types of talc (from Elementis Global, formerly Mondo Minerals Deutschland GmbH, Wuppertal, Germany) and one chalk grade (from Omya GmbH, Cologne, Germany) were used. The first talc grade has an average particle diameter (d50) of 4.5 µm and a specific surface area (Sm) of 6 m^2^/g, hereinafter denoted as “coarse talc”. The second grade with d50 of 2.2 µm and Sm of 10 m^2^/g will be described as “fine talc”. Only one fine type of chalk was used with a specific surface area (Sm) of 8 m^2^/g and a d50 of 1 µm. All data sheets do not provide information on a pretreatment.

### 2.2. Methods

#### 2.2.1. Sample Preparation

##### Blending

Polymer blends were extruded in a twin-screw extruder (ZSK 25, L/D ratio 40, Coperion GmbH, Stuttgart, Germany) at temperatures between 160 and 175 °C. Most of the blends have a ratio of PLA/PBS of 70:30. Additionally, a reverse ratio of PLA/PBS of 30:70, as well as another polymer combination by exchanging PBS with PBAT (PLA/PBAT: 70:30), was tested on both additional blends without adding inorganic fillers. Coarse talc, fine talc and chalk were added to the polymer blends in amounts of 5 and 15 phr (i.e., 5 or 15 parts of filler per hundred parts of polymer matrix, by weight). Processing parameters were kept constant for all compounds. An overview of the blend formulations and the purposes of the experimental series is given in Table 1.

##### Injection Molding

Specimens were prepared via injection molding on machinery type BA 600 CDC (Wittmann Battenfeld Deutschland GmbH, Meinerzhagen, Germany) within a temperature range of 160 to 190 °C. Both, tensile test rods (type 1 A) as well as step plates, were produced for each blend system. Step plates had three different thicknesses of 1, 1.5 and 2 mm, each with the same width and length dimensions of 30 and 60 mm. The plates were cut into three pieces, each with a uniform thickness of 1, 1.5 or 2 mm. Supplementary plates of 2 mm thickness were abraded manually by using sandpaper in order to assess the influence of surface roughness in the disintegration process.

##### Disintegration Trial

Disintegration trials were performed under defined lab conditions in accordance with the DIN EN ISO 16929 standard. The prepared plates were dried for 24 h at 40 °C in a drying chamber and weighed to determine their initial mass (m_1_). Boxes with a capacity of at least 1 L and 4 ventilation holes in the upper part, were filled with compost (degree of rotting 5, humidity > 50%, picked from the composting site “Asdonkshof” in Kamp-Lintfort, Germany). The boxes were filled with alternating layers of compost and test specimens. Thereby, all outer specimen surfaces were surrounded by compost and did not touch each other. To prevent cross-contamination, a separate box was used for each blend system. Per blend system, plates of three different thicknesses (1, 1.5 and 2 mm, the latter with smooth and rough surfaces) and tensile test rods were inserted. Boxes were stored in a climate chamber under conditions of 60 °C and 70% relative humidity, thus resembling industrial composting conditions in accordance with the DIN EN ISO 16929 standard. The following deviations from the standard are to be named:

Due to the limited space availability, single measurements (instead of double measurements) with differing ratios of test material to compost were used (approximately between 10 and 15% of the test material in relation to the amount of compost; standard: 1 or 10%). Sample sizes were different (30 × 60 mm^2^ instead of standard: 50 × 50 mm^2^). The temperature of the compost was not measured, since we used small boxes of 1 L volume in the temperature-controlled chamber. Aeration (10% oxygen concentration) was not checked but also assumed to be sufficient due to the internal ventilation rate and air exchange rate in the chamber. Instead, pH values were determined every 2 weeks to ensure a value above 5, as required in the standard.

#### 2.2.2. Sample Measurements

##### Degree of Disintegration

Determining the so-called degree of disintegration took place by evaluating weight differences of step plate specimens, present in three different thicknesses. The degree of disintegration *D_i_* was calculated by formula $\;D_{i}=\frac{m_{0}-m_{x}}{m_{0}}\cdot 100\;\left[\%\right]$.

For this, specimens were taken out of the boxes after defined time periods (weeks 0 to 4 once a week, weeks 5 to 12 every two weeks), cautiously cleaned from compost deposits under running water whilst lying on a 2 mm sized mesh sieve, dried at 40 °C for 24 h and weighed again (m_x_, where “x” means the week after collection and m_x_ is the mass of the dried specimen after the defined week of collection). Due to the sieve’s mesh width, fragments less than 2 mm size were washed away during the cleaning process and were not taken into account at weighing. In all cases, the determination of the degree of disintegration was based on single measurements.

##### Mechanical Properties

According to DIN EN ISO 527-2, tensile tests were measured (ZwickRoell 1474, Zwick Roell GmbH & Co. KG, Ulm, Germany) with the help of tensile bars (type 1 A). The determination took place on specimens without any disintegration time (reference materials) of each blend system and on specimens, collected after weeks 1, 2 and 3 after storage in compost under industrial composting conditions. After three weeks of storage time in the compost and later, the determination of the mechanical characteristics was no longer possible. The specimens revealed such a high degree of sample porosity that they already broke when clamped into the tensile testing machine. Therefore, we could no longer subject them to tensile stress. Mean values were calculated from five samplings each. They served to determine the influence of industrial composting conditions on mechanical properties due to degradation.

##### Differential Scanning Calorimetry (DSC)

Thermoanalytical properties of blend systems with and without undergoing disintegration were measured via differential scanning calorimetry (DSC 204 F1 Phoenix^®^, Netzsch Gerätebau GmbH, Selb, Germany). Approximately 10 mg was collected from step plates (of 2 mm thickness) after week 0 (reference materials, before disintegration) and after week 12 (to the end of disintegration trial). Each sample was measured using the same temperature profile (1st heating: −50 °C to 200 °C, holding time: 5 min, cooling: 200 °C to −50 °C, holding time: 5 min, 2nd heating: −50 °C to 220 °C, heating and cooling rates: 10 °C/min).

##### Microscopy

Specimens were inspected optically by using digital microscopy (VHX-6000, Keyence Deutschland GmbH, Neu-Isenburg, Germany). This served to record optical disintegration phenomena such as surface modifications—for example, fragmentation—and the accumulation of microorganisms on the specimens’ surfaces. The measurements were performed on samples of each blend system after defined disintegration periods. Additionally, scanning electron microscopy (SEM) (Vega 3, Tescan Orsay Holding a.s., Brno, Czech Republic) was used to inspect selected samples.

## 3. Results and Discussion

The results of the disintegration trials will be presented and discussed in comparison between the different samples and their behavior under composting conditions in each trial series. The measurement of disintegration by mass loss has to be regarded as a rather qualitative assessment. For example, there is an influence of the used compost, due to its different composition and quality, on the biodegradation behavior of polymer systems [32]. Next to such, the human factor has to be named as a potential source of error. Despite carefully conducting tests following a standard protocol, differences may occur caused by individual handling when collecting and washing the specimens. In some cases, a thorough cleansing leads to the loss of smaller fragments. In other cases, cleansing was difficult and compost residues remained on the samples increasing the weight measured. Furthermore, it has to be mentioned that the used biopolymers PLA, PBS and PBAT are known to be compostable according to the DIN EN ISO 14855 standard. These tests are performed with thin films or powdered material samples. The rate of disintegration of a plastic product is strongly dependent on the sample thickness. With the selected thick walled injection molded specimens, sufficiently long disintegration times were expected in order to assess differences between the materials tested in this study.

### 3.1. Influence of Blend Components after Composting Conditions

**Figure 1:**
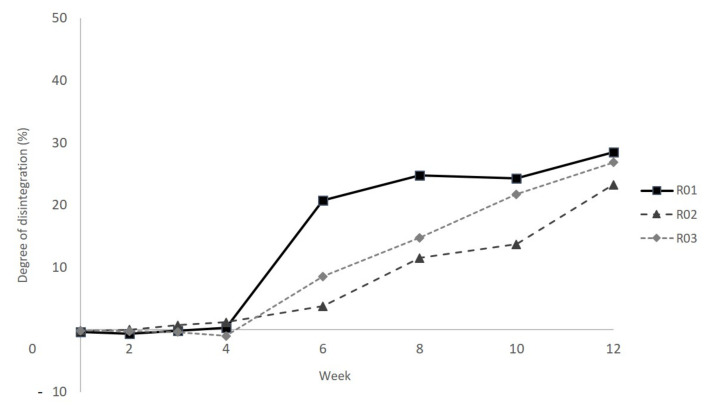
Influence of major and minor components of unfilled poly(lactic acid) (PLA)/poly(butylene succinate) (PBS), PBS/PLA and PLA/poly(butylene adipate-co-terephthalate) (PBAT) blend systems (ratio 7:3) on disintegration after defined times (measured on 2 mm thick plates). Compositions: R01: PLA/PBS (7:3); R02: PLA/PBS (3:7); R03: PLA/PBAT (7:3).

Comparing the polymer blend systems without filler loading, it seems that PLA has a higher degree of disintegration than PBS under industrial composting conditions (Figure 1). Using PLA as the major component (R01) leads to a pronounced increase in the degree of disintegration after week 4 of the composting period.

The combination of PLA and PBAT (R03) shows a similar behavior of a larger increase in degradation after week 4. This indicates that, after a first incubation period, PLA is characterized by a strong hydrolysis-induced degradation behavior under the conditions used. Once started, hydrolytic degradation in PLA proceeds faster in the inner molecular structure than on the outer surface. A self-catalyzing effect of carboxylic end groups formed upon chain cleavage leads to this kind of fast, self-induced and homogeneous ester hydrolysis, degrading the material in bulk [3,29]. Additionally, an erosion process on the specimens’ surfaces in the present biotic environment accelerated by enzymatic action is also to be expected [2,5,33].

PBS, as the major component in combination with PLA (R02), shows a smoother course of mass loss. The autocatalytic effect does not seem to be that pronounced as for PLA. On the one hand, Wang et al. also observed lower degradation rates of PBS in comparison to PLA [5]. On the other hand, and different from the results presented here, Zhang et al. showed in their work that a higher PBS content in a PLA/PBS blend system leads to a higher mass loss [34]. However, it needs to be mentioned that Zhang et al. used soil burial tests at lower temperatures. Meanwhile, it is well known that PLA, in particular, degrades much faster at higher temperatures [6,35]. On the other hand, it was published that the amount of PBS degrading microorganisms at 50 °C is higher than for PLA [2]. In case that this also holds true for the present study, where trials were conducted at 60 °C, conclusions that can be drawn are either that microbial PBS-degraders are not as effective as PLA-degraders or that PBS chain cleavage proceeds more slowly. Another explanation can be that the number of ester bonds in the PBS chain is lower compared to PLA. This allows fewer points of attack for degradation reactions.

**Figure 2:**
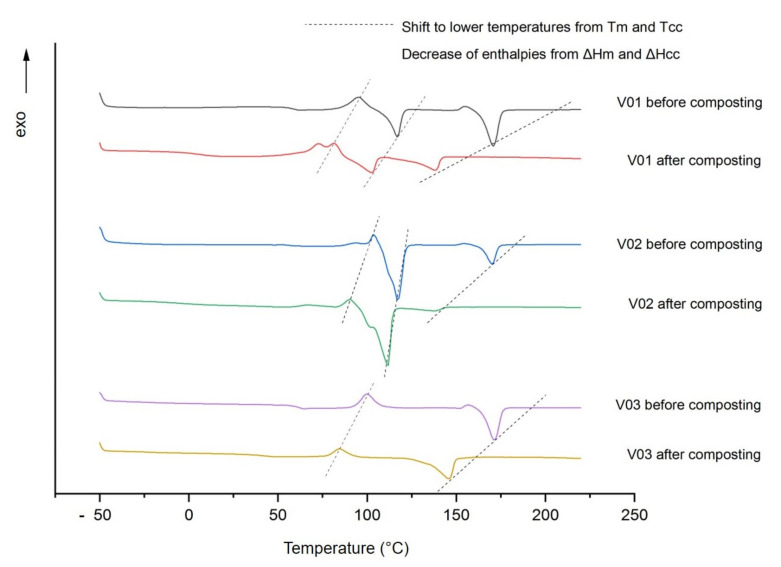
Differential scanning calorimetry (DSC) curves from unfilled blend systems before and after 12-week composting period. Compositions: R01: PLA/PBS (7:3); R02 PLA/PBS (3:7); R03: PLA/PBAT (7:3).

Figure 2 illustrates the second DSC heating curves of the blend systems R01 to R03 measured before and after the disintegration trials. In particular, data on glass transition temperatures T_g_, melting temperatures T_m_, melting enthalpy ΔH_m_ as well as cold crystallization temperatures T_cc_ and enthalpies ΔH_cc_ were compared. In general, all samples show a decrease in these characteristic temperatures and enthalpies towards lower values after composting. As polymers with lower molecular masses are associated with lower melting temperatures, this is a significant indicator for the occurring depolymerization process.

DSC thermograms of blend systems prior to composting revealed the characteristic temperature values for PLA, PBS and PBAT. T_g,PLA_ is at about 60 °C; T_m,PLA_ is at 170 °C. Mentioned for the sake of completeness, undetected T_g_ of PBS and PBAT used both fall in the range of −35 to −30 °C. Their melting points, T_m,PBS_ and T_m,PBAT_, are also almost equal: around 115 to 117 °C. After the composting period of 12 weeks investigated here, T_m,PLA_ decreases to 138 °C in PLA/PBS combinations, and to 146 °C for PLA/PBAT blends. An overview of measured values is present in Table 2.

In all cases, ΔH_cc PLA_ was not present after composting. Comparing PLA with PBS, the former showed a more significant decrease in melting temperature from 170 to nearly 138 °C. This stands in accordance with the above-discussed disintegration observations, in which the highest rate was attributed to PLA. In addition, the melt enthalpies of PLA after composting are lower than before. That indicates polymer chain cleavages obtained by hydrolytic and/or enzymatic degradation. In general, when comparing blends R01 and R02 before and after composting, it is noticeable that—with one exception—the melting enthalpies ΔH_m_ decrease after composting. Only PBS as major component (R02) is characterized by an increase in melting enthalpy after composting. Cho et al. found that, e.g., iso-crystallized samples (at 60 °C), formed spherulites, consisting of loosely packed but thicker fibrils than those of melt-quenched, non-isothermally crystallized samples [17]. Pivsa-Art et al. found out that the crystallization under isothermal conditions (measured at 90 °C) of pure PBS is completed much faster than that of PLA (6 min instead of 120 min). This suggests that the crystallization rate of PBS inhibits that of PLA. The degradation takes place in the crystalline region at a slower rate, which is confirmed by the lower degradation of R02 compared to R01 (see Figure 1) [36]. PBAT does not seem to have this pronounced suppressing effect on PLA melting enthalpy after composting (see R03 before and after composting, Figure 2).

### 3.2. Influence of Filler Type, Size and Amount

#### 3.2.1. Before Composting

When describing the general influence of fillers on PLA/PBS blends prior to composting, the effect on cold crystallization rates should be mentioned. In comparison to the unloaded PLA/PBS (70/30) blend, the use of fillers generally suppressed cold crystallization. This was particularly pronounced for PBS when using chalk (see Table 3). Instead of ΔH_cc, PBS_ of -17 J/g for the unfilled blend, it was reduced to −2.3 J/g by using 5 phr chalk and even to −1.7 J/g for 15 phr chalk. With the exception of the higher use of coarse talc, blending with both types of talc even completely prevented the cold crystallization effect. Talc thus seems to have a higher nucleation effect on PLA/PBS blends. This correlates with the statement of the research work of Pivsa-Art et al., who showed that talc promotes the crystallinity of PLA and acts as an inhibitor for crystallinity for PBS as well [36].

The influence of the filler types and amounts on the mechanical characteristics of the blend systems was also considered. The measured values before the composting period do not differ remarkably. The tensile strengths of the samples are all in the range between 50 and 57 MPa (see Table 4 week 0). For the non-disintegrated samples, and with regard to the tensile strength, a strong reinforcing effect due to the use of fillers on the blend system is therefore not detectable. Regarding Young’s modulus, reinforcement can be observed by the use of fillers. In particular, this applies to the coarse talc (3538 MPa, V07) and fine talc (3907 MPa, V08) compared to 2557 MPa for the reference ((R01), Table 4).

#### 3.2.2. Disintegration and Material Properties after 12 Weeks under Composting Conditions

**Figure 3:**
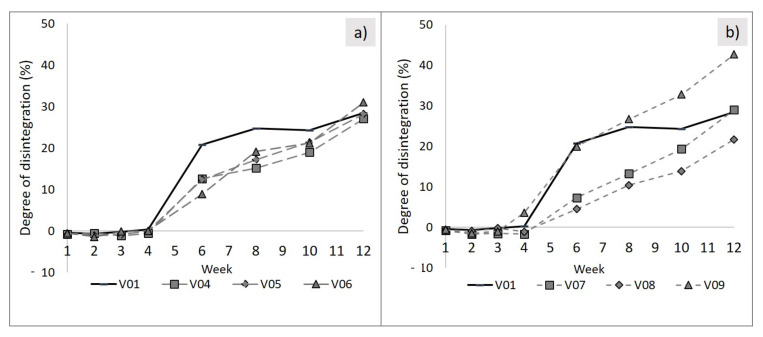
Influence of filler type and amount on the degree of disintegration, PLA/PBS (7:3) with 5 phr (**a**) and PLA/PBS with 15 phr content (**b**), measured on 2 mm thick plates. Compositions: R01: unfilled reference; V04/V07: plus 5/15 phr coarse talc; V05/V08: plus 5/15 phr fine talc; V06/V09: plus 5/15 phr chalk.

First, the influence of the fillers on the disintegration of the blends under industrial composting conditions shall be discussed. The influence of the different filler types has to be evaluated taking the content of the fillers into account. Using only a small amount of 5 phr of the filler materials, the degrees of disintegration are almost equal for all tested filler types (see left diagram, Figure 3). It seems that the use of fillers reduces the sharp increase between weeks 4 and 6 that was recorded for the unfilled blend (R01). Nevertheless, despite slower disintegration rates from weeks 4 to 10, nearly the same degree of disintegration was detected after the tests were completed (week 12).

In case of using higher filler amounts, disintegration curves are clearly different for the filler types examined (right diagram, Figure 3). Both talc types, coarse (V08) and fine (V09), seem to suppress the induced hydrolytic degradation of PLA rich blends. This can be recognized from the less pronounced slope of the curves around week 6. A similar effect was described in the literature for PLA samples combined with nanofillers. Fukushima et al. explained this effect with an increased crystallization rate which suppresses water absorption in the matrix [6].

However, using 15 phr chalk content, the same sudden increase in disintegration in week 6 was recorded as for the unfilled polymer blend. It can be concluded that hydrolytic degradation does not seem to be hindered. Additionally, the final value of disintegration is much higher than from the unfilled or the other filler loaded blend systems. The second largest degree of disintegration was observed for the use of 5 phr chalk (V06, Figure 3, left side). This underlines the thesis that chalk promotes degradation. A comparable effect was shown in an investigation of the degradation of neat PLA combined with clay minerals (at lower composting temperature conditions) [14,37]. From these results, Rapacz-Kmita et al. provided the thesis that the clay behaves as a kind of reservoir for water promoting hydrolytic processes in the bulk, which leads to an increased degradation [14].

The smallest degradation rate was recorded when using 15 phr of fine talc (V08, Figure 3, right side). This blend system (PLA/PBS: 7:3) became more stable against hydrolytic degradation.

Following DSC measurements and tensile tests help to assign the blend systems’ type of erosion. Table 3 presents an overview of measured data deduced from the second DSC heating.

**Figure 4:**
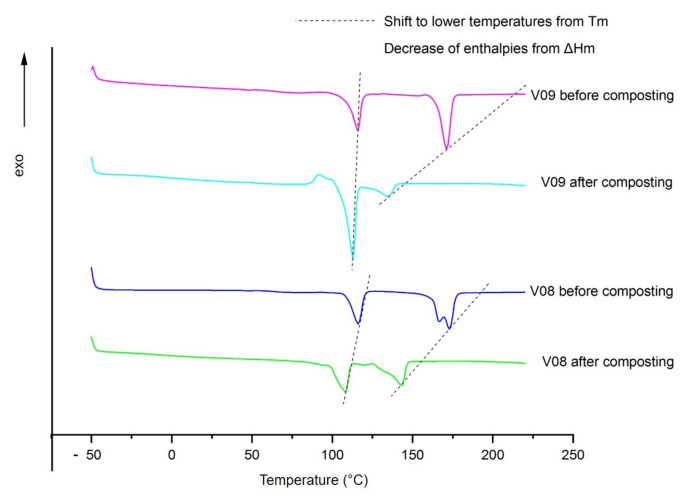
Exemplary DSC curves from filled blend systems before and after 12-week composting period. Compositions: PLA/PBS (7:3); V08: plus 15 phr coarse talc; V09: plus 15 phr chalk.

Exemplary heating curves from PLA/PBS blends (7:3) with 15 phr fine talc and chalk loading before and after 12 weeks of the industrial composting period are shown in Figure 4. As can be seen, talc loading, no matter if fine or coarse, leads to bimodal melting peaks of PLA. This corresponds to previous works, where a heterogeneous nucleation effect of talc on PLA crystals was explained [38]. Regardless of the talc type and content inserted, blend systems showed similar decreases of T_m_ and ΔH_m_ for PLA and PBS after composting. This corresponds to the shifts to lower temperatures observed for the unfilled blend systems, as described in the previous section (see Figure 1 and Figure 2). 

It was also observed that no cold crystallization peaks exist for both, PBS and PLA when using talc. On the other hand, chalk does not have such a significant suppressive effect (see Figure 4 and Table 3). Talc acts as a more effective nucleating agent for PLA/PBS blends, which leads to slower degradation rates. On the other hand, chalk influences the melting enthalpy of PBS after the composting period. Independent from the chalk content, ΔH_m,PBS_ experienced an increase from about 18 or 17 J/g (for 5 and 15 phr chalk, respectively) before composting up to 28 or 29 J/g (5 phr resp. 15 phr chalk) afterwards (see Table 3). The comparison with the values for the unfilled reference (ΔH_m,PBS_ 22 J/g before and 17 J/g after composting) also reveals that chalk acts as good nucleating agent for PBS after 12 weeks of composting. This outcome is explained by a kind of post-crystallization effect from PBS at industrial composting conditions, in which the prevailing temperatures of 60 °C are much higher than its Tg (−35 °C). Nevertheless, as onset and endset temperatures for samples before and after composting do not differ considerably, chain scission for PBS was not noted.

Furthermore, 15 phr chalk led to the highest difference in T_m_,_PLA_ and ΔH_m,PLA_. The initial value of 24 J/g melt enthalpy reduced to one-third, i.e., 8 J/g, after the 12-week composting period. The melt temperature decreased by about 36 °C, from 171.2 to 134.7 °C. This corresponds to the highest degree of disintegration, using a high content of chalk (see Figure 3).

**Figure 5:**
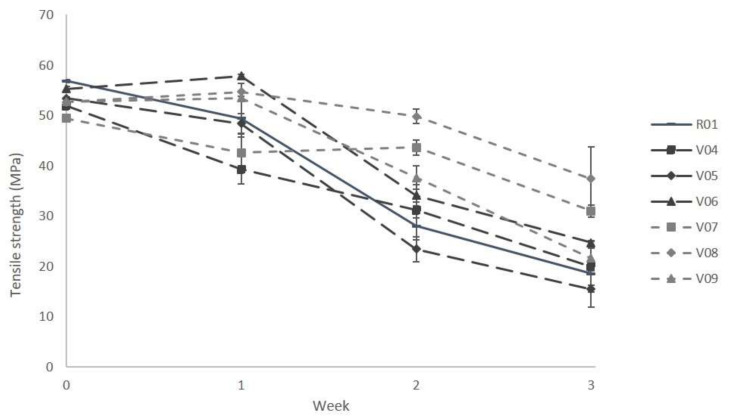
Tensile strength of PLA/PBS-based blend systems (7:3) loaded with 5 and 15 phr of coarse talc, fine talc and chalk, after 0 to 3 weeks located under industrial composting conditions. Compositions: R01: unfilled reference; V04/V07: plus 5/15 phr coarse talc; V05/V08: plus 5/15 phr fine talc; V06/V09: plus 5/15 phr chalk.

**Figure 6:**
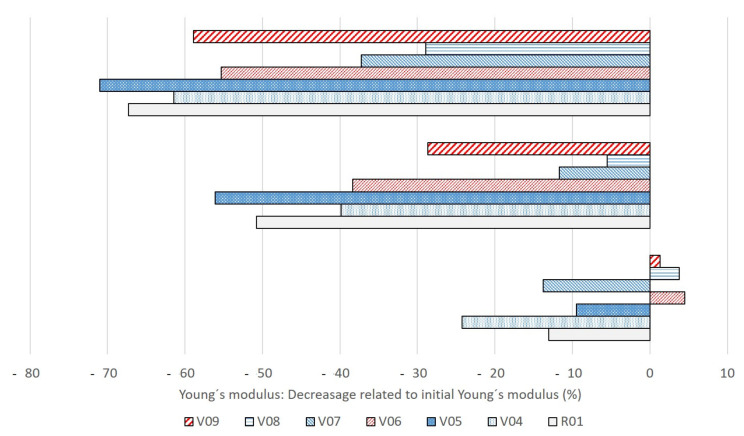
Percentage in loss of Young’s modulus of PLA/PBS-based blend systems (7:3) after 1, 2 and 3 weeks of storage under industrial composting conditions. Compositions: R01: unfilled reference; V04/V07: plus 5/15 phr coarse talc; V05/V08: plus 5/15 phr fine talc; V06/V09: plus 5/15 phr chalk.

Tensile tests were performed on specimens until week 3. Their results are depicted in Figure 5 and Figure 6. Samples with longer composting times could not be measured any more due to cracks and brittleness. Considerable changes in mechanical properties were detected from week 1 to week 3. This is prior to measurable mass losses as seen in Figure 3, which shows that for all samples no mass loss can be measured in the first four weeks of the composting period. If only a surface erosion process was present, no changes in mechanical properties or molecular mass would be measurable [3,10]. The decrease in mechanical strength preceding the quantifiable mass loss by erosion processes points at polymer degradation in the bulk caused by chain scissions and decreasing molecular mass.

After one week of storage under industrial composting conditions, the impact of filler loading on the change in mechanical properties is not that large. However, after two and three weeks, some trends can be identified. In general, decreasing strengths are recorded as expected for all specimens, no matter if the blend system is loaded with mineral fillers or not. The loss of mechanical properties is a consequence of the hydrolytic degradation [39]. In comparison with the unfilled reference blend, only the sample with 5 phr fine talc (V05) showed a larger relative decrease in tensile strength (about 70% decrease compared to 67% for the reference, cf. Figure 5). Compared with the reference (R01), in general, the use of the higher filler amount (15 phr) leads to a smaller decrease in tensile strength (V07 to V09, Figure 5) on one hand, but on the other hand to a higher reduction in Young’s modulus (V04 to V06, Figure 6). Using both talc types with 15 phr, the decrease in tensile strength from week 0 to week 3 was rather small (about 37 and 29% in comparison to 67% decrease in the reference material). This corresponds to the above-discussed disintegration rates, which were lowest using high amounts of fine and coarse talc (Figure 3). Taking a look at the mechanical property trends for chalk-filled samples (V06 and V09), a high decrease in strength can be noticed. The same applies to the measured Young’s modulus. For the three tested filler types with 15 phr content, only the chalk-filled blend (V09) yields a higher decrease than the reference (R01), whereas the use of talc resulted in a smaller relative decrease (see Figure 6). The results of the mechanical testing are well in line with the results of the discussed disintegration trial.

The trends can be explained by the surface treatment of the different filler materials and their specific surfaces with the largest value of 10 m^2^/g m^2^ in case of the fine talc used. The hydrophobic character of the filler surfaces hinders water absorption into the matrix material. A loading of 5 phr of fine talc is too small to reveal measurable effects, while apparently using 15 phr stabilizes the material against the decrease in mechanical properties. Previous studies using other fillers with high surface areas such as silicates showed similar effects. A lower decrease in mechanical properties as well as lower mass loss compared to unfilled systems were reported due to the use of such fillers [24,40]. The authors assume that next to the approach of hindered water absorption, this is due to microorganism intrusion being hindered and colonization inside the bulk.

**Figure 7:**
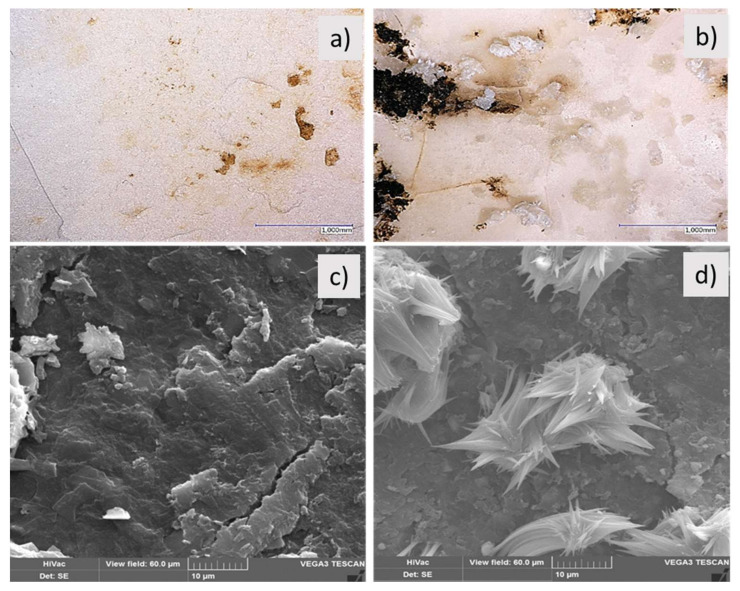
Comparison between fine talc (V08, (**a**,**c**)) and chalk (V09, (**b**,**d**)) loaded PLA/PBS (7:3) blend systems after 6 weeks ((**a**,**b**), **pictures above**) and 12 weeks ((**c**,**d**), **pictures below**) of disintegration under industrial composting conditions, each by loading 15 phr filler amount. Upper pictures using digital light microscopy, pictures below SEM photographs.

**Figure 8:**
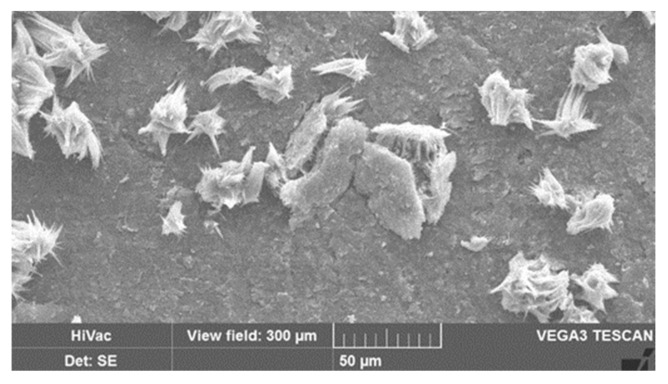
Calcium carbonate induced “crack formation” of loaded specimen (V09 after 8 weeks storage under industrial composting conditions).

In addition to measuring mechanical properties and the degree of disintegration, changes of the surface structure of the blend systems were investigated by microscopic examination. Not only did the rate of disintegration reveal differences regarding the use of chalk or talc, but also the sample surfaces appeared distinctively different (Figure 7). When using 15 phr of talc, only the formation of first surface cracks and discolorations as a result of the beginning decomposition after 6 weeks of composting can be noticed (exemplarily documented by showing the fine chalk type, left upper picture in Figure 7). After the same time, the use of chalk not only leads to crack formation, but also to the generation of microcrystals on the surfaces (right upper picture, Figure 8). These microcrystals consist of recrystallized chalk. In the bulk of the material, the polyester degrades by hydrolytical chain scission, which was proven by DSC and the decrease in mechanical properties. This mechanism goes along with the formation of acidic chain ends, which can react with water and the almost insoluble chalk to carbonic acid (H_2_CO_3_). We assume that chalk, generally insoluble in water but soluble in carbonic acid, is converted to calcium hydrogen carbonate (Ca(HCO_3_)_2_), which is more soluble in water [41,42]. At the material surface, in contact with the buffering compost moisture, calcium hydrogen carbonate can furthermore react with re-crystallized calcium carbonate. From the appearance of the crystals in the presented images (Figure 7, lower right picture), it can be concluded that the aragonite structure of calcium carbonate is formed, which can be expected because of the elevated temperatures (60 °C) in the experiments.

The formation of calcium carbonate on the surface also explains the higher grades of decomposition of the chalk-filled samples already described above (Figure 3). Because of its transfer to the surface, chalk-filled samples comprise an increasing number of voids, as can be seen in Figure 8. This leads to improved water penetration into the matrix, additionally accelerating hydrolytic degradation.

In contrast to this, corresponding with mechanical properties and as expected because of talc’s insolubility in water and acids, talc-filled samples do not show the formation of particles on the surface (Figure 7, lower left picture) and thus a considerably less void formation inside is assumed. Instead, due to its hydrophobic character, the use of talc leads to reduced access for water molecules into the polymer.

### 3.3. Influence of Sample Thickness 

**Figure 9:**
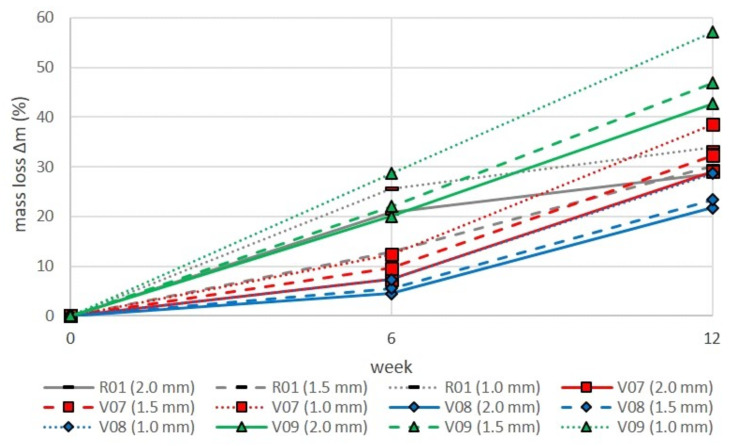
Sample mass loss in comparison of sample thickness and filler types due to industrial composting conditions. Compositions: R01: PLA/PBS blends (7:3) unfilled; V07: plus 15 phr coarse talc; V08: plus 15 phr fine talc; V09: plus 15 phr chalk.

With one exception, a nearly linear relationship can be seen regarding the influence of sample thickness on the degradation rate. Figure 9 shows the percentage of mass loss of specimens with different thicknesses after 6-and 12-week composting periods in relation to their initial masses. Unfilled PLA/PBS blend systems (R01, Figure 9) were measured as well.

In the first 6 weeks, the unfilled blends showed a strong increase in mass loss. With the exception of the value for the 1.5 mm thick reference plate after 6 weeks, which is regarded as a measurement outlier, an increase in degradation with lower specimen thickness is recorded.

In direct comparison with the unfilled blend system (R01), all tested fillers influenced the disintegration behavior. It can be seen that both talc types led to a progressive rise of mass loss between weeks 6 and 12 of composting. Instead, the use of chalk yields a linear and very steep degradation progression during these composting tests. With a total of 28.7% mass loss after week 6 and 57.1% after week 12, both values for 1 mm plate thickness, chalk leads to a much higher, almost twofold disintegration of the blend systems compared to the unfilled blends. This is consistent with the previous statement that chalk facilitates water penetration into the bulk. Thereby, bulk erosion is promoted. The relation between sample thickness, composting time and mass loss can be the basis for predefining the disintegration behavior of PLA/PBS blend systems under industrial composting conditions using an appropriate degree of chalk loading.

Although there is no such clear dependency of disintegration rate from talc filler loading, a general trend can be seen. The progressive increase in the disintegration curve can be interpreted by the hindered water absorption during the first few weeks. In the first few weeks, the hydrophobic talc filler causes reduced water penetration into the bulk of the filled samples.

### 3.4. Influence of Surface Roughness

**Figure 10:**
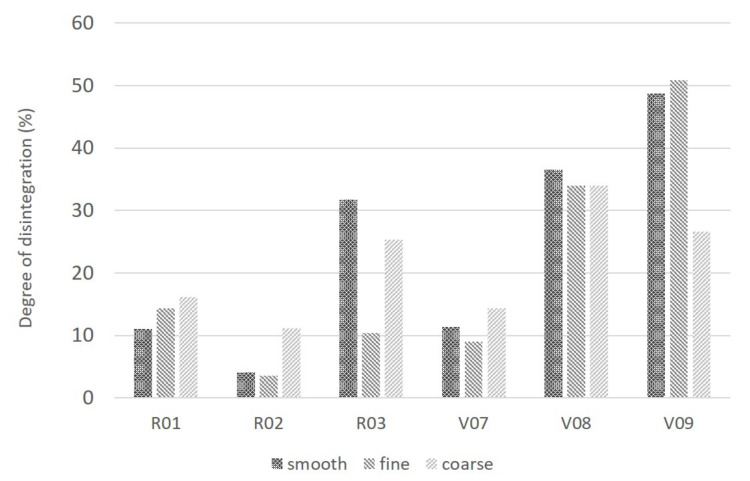
Comparison between sample surface structures and degree of disintegration, measured after 12-week composting period. Compositions: R01: PLA/PBS (7:3); R02: PBS/PLA (7:3); R03: PLA/PBAT (7:3); V07: PLA/PBS (7:3) plus 15 phr coarse talc; V08: PLA/PBS (7:3) plus fine talc; V09: PLA/PBS (7:3) plus chalk.

Whether the structures of the sample surfaces have an influence on polymer degradation or not, e.g., due to enzymatic or abiotic processes, was evaluated as well. Therefore, specimens were abraded (in two different grades) or smoothed and the disintegration rate after 12 weeks of composting under industrial conditions was determined. Figure 10 gives an overview of the degree of disintegration of specimens with different surface roughnesses. Even though a slight increase in degree of disintegration with increasing roughness is visible for the reference material (R01), no clear trend can be seen for any of the other samples. Hence, the roughness of the sample surface does not seem to have a decisive influence of the decomposition rate. This is another hint to the prevailing bulk erosion mechanism for the polymers and test conditions under consideration.

## 4. Conclusions

In summary, our analyses of disintegration experiments of PLA/PBS blends indicate that the degradation process of all the materials tested proceeds in the bulk of the specimens. Additionally, it can be concluded that the use of the mineral fillers—chalk and talc—significantly influences the disintegration behavior under industrial composting conditions. Based on these findings, a targeted control of the disintegration rate can generally be regarded as possible. Ultimately, it seems possible to come to predictions of disintegration times with a dependence on sample thickness, polymer composition, filler type and loading. However, it is necessary to evaluate this assumption in systematic follow-up studies to evaluate and consolidate the discussed results. This work provides a first small contribution to this theory.

## Figures and Tables

**Table 1:** Blend formulations and experimental purposes.

Trial Number	PLA	PBS	PBAT	Talc Coarse	Talc Fine	Chalk	Experimental Purpose	
R01	70	30					References	unfilled blend
R02	30	70						influence of major component
R03	70		30					influence of minor component
V04	70	30		5			influence of particle size	
V05	70	30			5			influence of filler type
V06	70	30				5		
V07	70	30		15			influence of (higher) filler amount	
V08	70	30			15			
V09	70	30				15		

**Table 2:** Thermal values from unfilled blend systems before and after composting.

	T_g,PLA_ [°C]	T_cc_,_PBS_/ T_cc,PBAT_ [°C]	ΔH_cc_,_PBS_/ ΔH_cc_,_PBAT_ [J/g]	T_cc_,_PLA_ [°C]	ΔH_cc_,_PLA_ [J/g]	T_m_,_PBS_/ T_m_,_PBAT_ [°C]	ΔH_m_,_PBS_/ ΔH_m_,_PBAT_ [J/g]	T_m_,_PLA_ [°C]	ΔH_m_,_PLA_ [J/g]
R01_w0	58.6	95.0	−17.04	154.9	2.6	116.8	21.9	170.7	31.6
R01_w12	6.5	72.7; 81.2 *	−33.54	n.d.	n.d.	102.4	17.0	138.0	20.4
R02_w0	59.2	103.6	−8.14	154.7	-1.5	117.1	50.9	169.9	13.3
R02_w12	n.d.	90.3	−6.14	n.d.	n.d.	111.4	59.3	137.6	4.8
R03_w0	61.4	99.9	−17.73	156.7	−2.1	n.d.	n.d.	171.2	27.7
R03_w12	42.2	84.3	−11.82	n.d.	n.d.	n.d.	n.d.	145.9	31.6

*: bimodal curve; w0: before composting period; w12: after 12-week composting period; n.d.: not detected. Compositions: R01: PLA/PBS (7:3); R02: PLA/PBS (3:7); R03: PLA/PBAT (7:3).

**Table 3:** Thermal values of mineral-filled blend systems before and after composting.

	T_g,PLA_ [°C]	T_cc_,_PBS_ [°C]	ΔH_cc_,_PBS_ [J/g]	T_cc_,_PLA_ [°C]	ΔH_cc_,_PLA_ [J/g]	T_m_,_PBS_ [°C]	ΔH_m_,_PBS_ [J/g]	T_m_,_PLA_ [°C]	ΔH_m_,_PLA_ [J/g]
R01_w0	58.6	95.0	−17.0	154.9	−2.6	116.8	21.9	170.7	31.6
V04_w0	n.d.	n.d.	n.d.	n.d.	n.d.	116.5	25.6	163.3; 172.4 *	31.7
V04_w12	n.d.	n.d.	n.d.	n.d.	n.d.	105.1	17.2	139.4	16.8
V05_w0	n.d.	n.d.	n.d.	n.d.	n.d.	116.4	23.7	165.5; 172.4 *	31.7
V05_w12	n.d.	n.d.	n.d.	n.d.	n.d.	106.4	17.4	140.7	18.9
V06_w0	63.7	91.7	−2.3	157.3	-0.8	115.9	17.7	171.1	26.8
V06_w12	n.d.	88.8	−3.3	n.d.	n.d.	109.7	28.4	140.9	19.0
V07_w0	n.d.	94.0	−1,1	n.d.	n.d.	116.2	17.4	165.4; 172.2*	25.6
V07_w12	n.d.	n.d.	n.d.	n.d.	n.d.	107.6	13.6	143.7	17.0
V08_w0	n.d.	n.d.	n.d.	n.d.	n.d.	116.0	16.1	166.6; 172.9*	27.4
V08_w12	n.d.	n.d.	n.d.	n.d.	n.d.	108.3	17.2	143.2	16.8
V09_w0	n.d.	94.5	−1.7	158.7	−0.7	116.0	17.0	171.2	23.6
V09_w12	n.d.	92.0	−9.2	n.d.	n.d.	113.1	29.2	134.7	7.9

Legend: w0: before composting period; w12: after 12-week composting period; n.d.: not detected; Compositions: R01: unfilled reference; V04/V07: plus 5/15 phr coarse talc; V05/V08: plus 5/15 phr fine talc; V06/V09: plus 5/15 phr chalk.

**Table 4:** Young’s modulus and tensile strength of tensile test specimen before and after 1, 2 and 3 weeks of composting.

	Week 0	Week 1	Week 2	Week 3
	Young’s modulus [MPa]			
R01	2557 ± 43	3155 ± 39	3230 ± 90	3226 ± 104
V04	2817 ± 148	3259 ± 60	3523 ± 86	3765 ± 51
V05	2929 ± 106	3457 ± 73	3523 ± 92	3960 ± 131
V06	2561 ± 168	3128 ± 38	3099 ± 76	3438 ± 83
V07	3538 ± 343	3647 ± 114	4113 ± 127	4460 ± 205
V08	3907 ± 189	4261 ± 70	4470 ± 176	4823 ± 200
V09	2718 ± 148	3144 ± 159	3380 ± 61	3505 ± 46
	Tensile strength [MPa]			
R01	56.9 ± 0.3	49.5 ± 3.7	28.0 ± 2.7	18.6 ± 2.4
V04	51.9 ± 0.3	39.3 ± 3.0	31.2 ± 1.7	20.0 ± 5.1
V05	53.4 ± 0.2	48.4 ± 2.0	23.4 ± 2.4	15.5 ± 3.6
V06	55.3 ± 0.4	57.8 ± 0.4	34.1 ± 2.1	24.7 ± 0.5
V07	49.4 ± 0.5	42.6 ± 3.8	43.7 ± 1.5	31.0 ± 1.2
V08	52.7 ± 0.4	54.7 ± 1.7	49.8 ± 1.4	37.5 ± 6.4
V09	52.8 ± 0.2	53.5 ± 0.3	37.7 ± 2.4	21.7 ± 1.9

Compositions: R01: unfilled reference; V04/V07: plus 5/15 phr coarse talc; V05/V08: plus 5/15 phr fine talc; V06/V09: plus 5/15 phr chalk.
